# Quality of Life and Stress-Related Psychological Distress Among Families Caring for Children with Cardiac Malformations Under Conservative Treatment: A Cross-Sectional Study Using the 36-Item Short Form Health Survey, the Perceived Stress Scale, and the Parental Burnout Assessment Scale

**DOI:** 10.3390/diseases13040095

**Published:** 2025-03-25

**Authors:** Andrada Ioana Dumitru, Mirabela Dima, Marioara Boia

**Affiliations:** 1 Doctoral School, “Victor Babes” University of Medicine and Pharmacy Timisoara, 300041 Timisoara, Romania; andrada.dumitru@umft.ro; 2 Discipline of Neonatology, “Victor Babes” University of Medicine and Pharmacy Timisoara, 300041 Timisoara, Romania; boia.marioara@umft.ro

**Keywords:** quality of life, congenital disease, cardiology, pediatrics

## Abstract

Families caring for children with congenital cardiac malformations under conservative management frequently experience psychological distress, which can compromise their overall quality of life (QoL). Despite growing recognition of the psychosocial burdens these families face, few studies have quantitatively assessed their QoL and stress-related outcomes. We aimed to evaluate the QoL, perceived stress, and parental burnout in caregivers of pediatric patients with cardiac malformations under conservative treatment. Methods: We conducted an observational, cross-sectional study of 78 caregivers (median age of 36 years) whose children (median age was 6.0 months) received conservative management for congenital cardiac malformations. Data were collected at two time points (diagnosis of congenital disease approximately at the time of birth, and six months after diagnosis) using the 36-Item Short Form Health Survey (SF-36), the Perceived Stress Scale (PSS), and the Parental Burnout Assessment (PBA). Statistical analyses included paired t-tests, chi-square tests, and Pearson correlation analyses; *p*-values < 0.05 were considered statistically significant. Results: Mean SF-36 Physical Component Summary scores significantly increased from 59.7 ± 11.7 at baseline to 63.5 ± 12.1 at six months (*p* = 0.026). PSS scores decreased from 22.9 ± 6.2 to 20.4 ± 5.9 (*p* = 0.012), indicating reduced perceived stress. Parental Burnout Assessment total scores also declined from 44.9 ± 8.5 to 40.1 ± 8.0 (*p* = 0.003). Correlation analyses revealed moderate negative correlations between SF-36 domains and both PSS (r range: −0.40 to −0.58) and PBA (r range: −0.34 to −0.52). Conclusions: Our findings highlight the multifaceted challenges faced by caregivers of children with cardiac malformations under conservative treatment. Improvements in QoL, accompanied by decreased perceived stress and parental burnout over six months, underscore the potential value of both clinical monitoring and targeted psychosocial support. Future research should explore larger, multicenter cohorts and longer follow-up durations to clarify long-term trends. Implementing interventions aimed at alleviating stress and burnout in these families may be pivotal for sustaining well-being and enhancing patient outcome.

## 1. Introduction

Congenital cardiac malformations are among the most common birth defects worldwide, affecting approximately 8 to 12 infants per 1000 live births, with wide variations based on geographic and population-based factors [1,2].

While surgical interventions have greatly improved survival rates, many children remain under conservative management due to the complexity or type of malformation, parental choice, or medical constraints [3]. In this context, caregivers shoulder considerable burdens associated not only with medical follow-up and day-to-day care but also with the psychological and emotional weight of uncertainty.

Conservative treatment for cardiac malformations often involves regular clinical evaluations, cardiac imaging, and ongoing medication regimens to ensure a stable hemodynamic status [4]. Unlike surgical correction, which can provide partial or complete anatomical solutions, conservative management may place caregivers in prolonged waiting periods. These extended timelines can foster anxiety about future interventions, disease progression, or complications [5]. Consequently, stress-related psychological distress emerges as an essential domain for investigation in families managing pediatric chronic conditions.

Quality of life (QoL) has emerged as a critical measure in pediatric cardiology, wherein children’s health outcomes are closely tied to family functioning and caregiver well-being [6]. Recent evidence suggests that parental stress, the burden of caregiving tasks, and burnout can detrimentally affect not only the caregiver’s mental health but also the child’s adherence to treatment and overall development [7].

The 36-Item Short Form Health Survey (SF-36) is widely used to capture multidimensional aspects of QoL, including physical functioning, bodily pain, general health, vitality, social functioning, role limitations, and mental well-being [8]. Meanwhile, the Perceived Stress Scale (PSS) is a validated tool to assess subjective stress levels, while the Parental Burnout Assessment (PBA) specifically evaluates the exhaustion and emotional distance caregivers may experience in relation to their parental role, which was also validated in Romania [9,10,11]. Although these instruments have been employed in separate contexts, their combined use provides a holistic view of family well-being in a pediatric cardiology setting. Moreover, there is a scarcity of research contrasting stress and burnout across various demographic subgroups, such as maternal vs. paternal roles, younger vs. older caregivers, or families of children with mild vs. severe malformations. Addressing these gaps is critical for optimizing family-centered care models in pediatric cardiology [12,13].

Accordingly, the objectives of this study were two-fold. First, we sought to assess changes in caregiver QoL, perceived stress, and parental burnout over a six-month period among families whose children have cardiac malformations managed conservatively. Second, we aimed to investigate the relationships among these psychosocial variables and demographic or clinical factors. Our overarching goal was to identify potential areas for targeted intervention to bolster caregiver well-being and thereby improve both short- and long-term child outcomes.

## 2. Materials and Methods

### 2.1. Study Design and Ethical Considerations

This study was designed as a prospective analysis of families caring for children with congenital cardiac malformations managed without definitive surgical correction. Data collection was performed at two time points (baseline and six-month follow-up) between May 2023 and December 2024 at the Timiş County Emergency Clinical Hospital “Pius Brinzeu”. Ethical approval was obtained from the institutional review board (IRB) of the hospital (Approval ID: 392 from 25 April 2023), in accordance with the Declaration of Helsinki. Written informed consent was obtained from all caregivers before enrollment, emphasizing confidentiality and voluntary participation.

All medical data (child’s diagnosis, disease severity, follow-up regimen) and caregiver data (age, gender, educational level, familial composition) were de-identified and stored securely. Inclusion criteria consisted of (1) primary caregivers (parents or legal guardians) of children aged 1–12 years with a confirmed congenital cardiac malformation, (2) conservative or medical management without imminent plans for surgical intervention, (3) caregivers with first child, and (4) willingness to complete standardized questionnaires at both study intervals. Exclusion criteria included (1) caregivers of children scheduled for cardiac surgery within three months, (2) those with insufficient language proficiency, and (3) caregivers with significant chronic diseases such as diabetes, chronic obstructive pulmonary disease, chronic kidney disease, cancer, severe asthma, autoimmune diseases like rheumatoid arthritis or lupus, and major neurological disorders such as multiple sclerosis.

At the outset, we performed a formal power analysis using G*Power (Version 3.1) to estimate the minimum number of participants required to reliably detect moderate effects (Cohen’s d = 0.5) in our primary outcomes, with α set at 0.05 and a power of 0.80. Based on these assumptions and accounting for an expected attrition rate, we determined that a sample size of 60 caregivers would be sufficient to meet our study objectives. Ultimately, 78 individuals completed both assessments, exactly aligning with our pre-specified target and helping ensure that our analyses were adequately powered.

**Figure 1:**
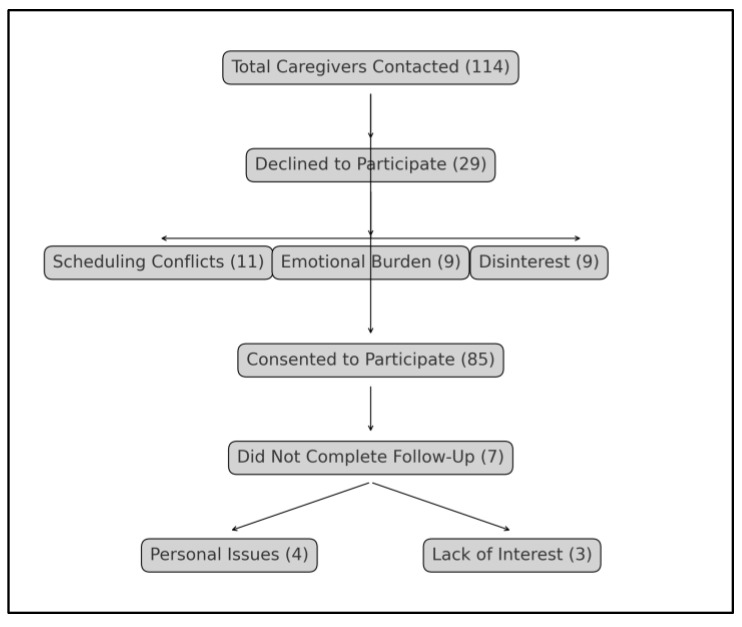
Flowchart of participants’ inclusion.

A total of 114 potential caregiver participants were initially contacted. Of these, 29 declined to participate, with reasons cited including scheduling conflicts (11 caregivers), concerns about the emotional burden of study questions (9 caregivers), and disinterest in research participation (9 caregivers). This left 85 caregivers who consented to participate. However, during the course of the study, 7 participants did not complete the necessary follow-up measurements due to unforeseen personal issues (4 caregivers) and lack of further interest (3 caregivers). Ultimately, the study concluded with 78 caregivers who completed all required baseline and follow-up measurements (Figure 1).

### 2.2. Measures and Instruments

Caregivers completed the SF-36, which evaluates eight domains of QoL, yielding Physical and Mental Component Summary scores. The SF-36 has been validated in multiple populations and demonstrates robust internal consistency (Cronbach’s alpha generally > 0.80). Higher domain scores reflect better QoL. The Perceived Stress Scale (PSS) measured subjective stress over the preceding month. Caregivers rated each item on a 5-point scale (0 = “never” to 4 = “very often”), producing a total score (range: 0–40). The Parental Burnout Assessment (PBA) evaluated fatigue, emotional distancing, and a sense of ineffectiveness specific to parenting roles. It yields a global burnout score, with higher values indicating greater burnout risk.

Child-specific clinical data included the type of cardiac malformation (e.g., ventricular septal defect, atrial septal defect, tetralogy of Fallot, or complex pathologies), severity classifications (mild, moderate, severe), and scheduled follow-up intervals. Family demographic variables included caregiver age, gender, education, and whether the caregiver was the mother, father, or another relative. All questionnaires were administered by trained research staff in a private setting, typically requiring 20–30 min for completion.

### 2.3. Data Collection and Management

At baseline, caregivers were approached in the inpatient pediatric cardiology clinic or during routine follow-up outpatient visits. A dedicated research coordinator explained the study objectives and addressed any questions. Once consent was given, participants completed the SF-36, PSS, and PBA instruments, along with a demographic form. Medical data about the child’s condition were extracted from electronic health records by a pediatric cardiologist on the research team.

The clinical monitoring of caregivers and their children was conducted bi-monthly by a team of pediatric cardiologists and specialized nurses, ensuring consistent assessment of the child’s health and the caregiver’s well-being. Psychological support was provided through monthly sessions conducted by licensed psychologists, which included cognitive–behavioral therapy and stress management techniques tailored specifically for caregivers of children with cardiac malformations.

Six months later, participants were contacted via telephone and asked to return for a follow-up assessment coinciding with the child’s routine cardiac evaluation. This timing minimized additional burdens on families. Data were systematically stored in an encrypted database accessible only to designated study personnel. Prior to analysis, all records were reviewed for completeness and consistency; incomplete questionnaires were excluded from final analyses.

### 2.4. Statistical Analysis

Data were analyzed using SPSS version 27.0 (IBM Corp., Armonk, NY, USA). Descriptive statistics included means, standard deviations, medians, and interquartile ranges. Normality was evaluated through the Shapiro–Wilk test. For normally distributed continuous outcomes (e.g., SF-36 summary scores, PSS total, PBA total), paired t-tests compared baseline vs. follow-up scores. If normality was violated, the Wilcoxon signed-rank test was utilized. Categorical variables (e.g., child’s severity group) were examined with chi-square or Fisher’s exact tests as appropriate.

We used Pearson’s correlation coefficients to investigate the relationships among the SF-36 summary scores, PSS totals, and PBA totals. Before applying Pearson’s correlation, we evaluated the normality of the SF-36, PSS, and PBA total scores using both the Shapiro–Wilk test and visual inspection of Q–Q plots. We performed subgroup comparisons of the child’s malformation severity (mild vs. moderate vs. severe), caregiver’s gender (mother vs. father), and educational level (high school vs. higher education). The subgroup analyses were adjusted to test multiple hypotheses by the Bonferroni–Holm method. Significance was set at *p* < 0.05, two-tailed. Effect sizes were reported where relevant to facilitate interpretation. A pos hoc power analysis was carried out, indicating adequate power (0.80) for detecting medium effect sizes in paired analyses. ANCOVA models with interactions and adjustment for baseline values were performed.

## 3. Results

A total of 85 caregivers were initially enrolled. Seven caregivers were lost to follow-up, resulting in a final sample of 78 for analysis. Table 1 summarizes the demographic profile of the 78 caregivers who completed this study. The median caregiver age was 36 years, with a distribution skewed slightly toward younger adults in their early thirties to mid-forties. Notably, there was a higher proportion of female caregivers (71.8%) compared to males (28.2%). Educational levels varied, with 39.7% holding a high school degree as their highest qualification and 60.3% reporting college or university-level education. The children’s mean age was 5.9 ± 2.2 months, and the gender split among children was 44 males vs. 34 females. Regarding the severity of cardiac malformation, nearly half of the children had moderate conditions (48.7%), while about one-fifth faced severe malformations (20.5%).

Table 2 displays the mean SF-36 scores at the baseline and after six months. Statistically significant improvements emerged in several domains: Physical Functioning (*p* = 0.034), General Health (*p* = 0.044), Vitality (*p* = 0.028), and Mental Health (*p* = 0.036). Although some domains like the Physical Role and Bodily Pain demonstrated numerical improvements, they did not reach statistical significance (*p* > 0.05). The Physical Component Summary score (PCS) increased from 59.7 ± 11.7 to 63.5 ± 12.1 (*p* = 0.026), indicating a small-to-moderate effect size. Notably, the Mental Component Summary (MCS) score also rose, but without statistical significance (*p* = 0.056).

Table 3 summarizes the changes in perceived stress (PSS) among caregivers from the baseline to six-month follow-up. The total PSS score showed a statistically significant decrease, dropping from 22.9 ± 6.2 to 20.4 ± 5.9 (*p* = 0.012).

Table 4 details the parental burnout dimensions at the baseline and at six months. Notably, all three domains—Exhaustion in Parenting, Emotional Distancing, and Loss of Parental Fulfillment—demonstrated statistically significant improvements (*p* < 0.05). The Total PBA score decreased from 44.9 ± 8.5 to 40.1 ± 8.0 (*p* = 0.003). The most pronounced shift was observed in the “Loss of Parental Fulfillment” subscale, which showed a mean change of approximately 2.2 points (*p* = 0.004).

**Figure 2:**
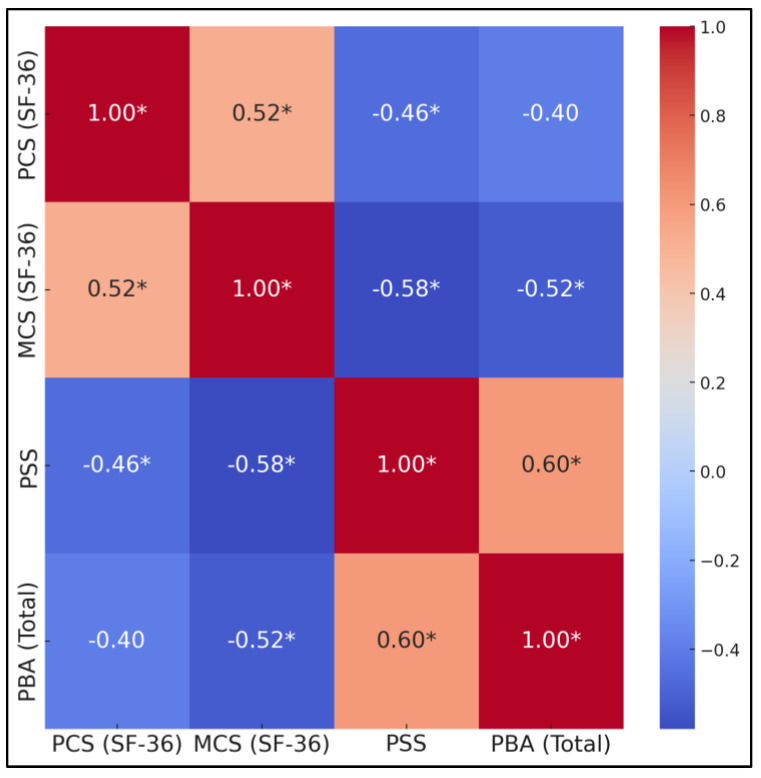
Correlation heatmap of follow-up values. * statistically significant (*p*-value < 0.05).

Table 5 and Figure 2 illustrate the interrelations among the SF-36 Physical Component Summary (PCS), SF-36 Mental Component Summary (MCS), PSS total, and PBA total at the six-month follow-up. Statistically significant correlations emerged in all tested relationships. Specifically, PCS and MCS scores were moderately positively correlated (r = 0.52), indicating that caregivers who felt physically better also tended to report better mental well-being. Conversely, both PCS and MCS were negatively and moderately correlated with PSS (r = −0.46 and r = −0.58, respectively). The negative correlations were between PCS, MCS, and PBA (r range: −0.40 to −0.52). Notably, PSS and PBA were highly positively correlated (r = 0.60).

Table 6 presents the subgroup analysis examining changes in SF-36 PCS, PSS, and PBA by the severity of the child’s cardiac malformation and caregiver gender. Among families dealing with mild or moderate malformations, improvements in PCS were statistically significant (*p* < 0.05). For caregivers facing severe malformations, although numerical improvements existed, statistical significance was not achieved (*p* > 0.05). Regarding perceived stress (PSS) and parental burnout (PBA), a similar pattern emerged: caregivers in the mild and moderate groups showed significant reductions in both stress and burnout, while those in the severe group reported improvements that did not reach significance. In terms of gender, female caregivers demonstrated statistically significant gains in PCS (*p* = 0.031) alongside notable decreases in PSS (*p* = 0.014) and PBA (*p* = 0.008).

## 4. Discussion

### 4.1. Literature Findings

In this observational study, we investigated the quality of life, perceived stress, and parental burnout among caregivers of children with cardiac malformations under conservative treatment. Our findings demonstrate that over a six-month period, caregivers reported modest but statistically significant improvements in several SF-36 domains, reduced perceived stress, and lowered burnout levels. These results extend prior evidence indicating that a child’s stable or predictable clinical status can facilitate better coping strategies and adaptation in caregivers [14,15]. The gradual nature of conservative treatment, which involves routine check-ups and steady monitoring, may also provide time for families to develop social and psychological resources, thereby reducing stress and exhaustion. On the other side, this observed change may be as much due to the natural rebound in mental well-being after a severe shock, even when there are no mitigating factors or circumstances.

Moreover, reduced uncertainty about the child’s immediate clinical status or improved coping mechanisms over time may have contributed to lowering stress. Given that the PSS captures general perceptions of stress rather than disease-specific anxiety, these results may also reflect improvements in overall life circumstances, such as better financial planning or emotional support from extended family. Nonetheless, a mean score still above 20 indicates that many caregivers continue to face considerable stress, warranting further psychosocial interventions. These findings reinforce the notion that while a significant proportion of families adapt over time, additional support measures—such as group counseling or stress management programs—could provide a further benefit.

The moderate to strong correlations observed among the SF-36 domains, PSS, and PBA suggest an interdependent relationship, where improvements in one domain potentially reinforce gains in others. These findings align with earlier research linking physical well-being and mental health in caregivers of pediatric patients with chronic conditions [16,17]. Moreover, the subgroup analysis underscores that family experiences are not uniform; disease severity and caregiver gender can influence the trajectory of QoL and stress outcomes. The less pronounced improvements observed in severe malformation cases may reflect persistent uncertainty or clinical challenges that overshadow typical coping mechanisms [18]. Similarly, the more robust gains among female caregivers might be due to their more frequent engagement with healthcare services or additional psychosocial support resources.

The emotional and developmental repercussions of congenital heart disease (CHD) on both parents and children have been pivotal areas of investigation. A study conducted by Charles Lepage et al. [19] delved into how parenting stress during infancy influenced neurodevelopmental outcomes at 24 months in children with CHD. Their findings revealed significant correlations between elevated levels of parenting stress and diminished outcomes in cognitive, receptive language, and gross motor skills, explaining between 14% and 18% of the variance in these areas. Conversely, this stress did not significantly affect expressive language or fine motor skills. In a similar vein, a qualitative study by Erica Sood et al. [20] focused on the unique stress experiences of mothers and fathers when caring for a young child with CHD. This study emphasized the differing responses between genders, with fathers particularly noting the stress of not being able to shield their child from the pain of surgery and the challenges of maintaining employment while supporting their family during such critical times. Fathers tended to utilize workplace support more than hospital-based or peer-to-peer CHD supports, which contrasted with mothers’ experiences.

Similarly, the studies conducted by Astrida Kaugars et al. and Nadya Golfenshtein et al. provide insights into the significant and enduring stress faced by parents of children with CHD. Kaugars et al. [21] observed that among parents of children aged 3 to 13, those with children with a single-ventricle anatomy reported clinically significant levels of stress, with these parents facing more frequent and severe illness-related stress compared to parents of children with a two-ventricle anatomy. Meanwhile, Golfenshtein et al. [22] documented that parents of infants with CHD consistently reported higher stress levels across the first year of life, with stress particularly pronounced on the demandingness subscale and life stress subscale at 12 months. Specifically, parents of CHD infants had significantly higher scores on the Parenting Stress Index than parents of healthy infants, indicating a sustained high stress level that impacts their coping mechanisms over time.

Woolf-King et al. [23] conducted a systematic review, identifying that parents of children with critical congenital heart defects (PCCHDs) face significant mental health challenges, with up to 30% exhibiting symptoms consistent with posttraumatic stress disorder and 25% to 50% reporting elevated symptoms of depression and anxiety. Notably, between 30% and 80% of these parents experienced severe psychological distress, indicating widespread and substantial mental health morbidity among this population. In a similar manner, the study by Roberts et al. [24] found that a significant proportion of parents of infants with CHD also reported moderate to severe mental health issues, with 25% experiencing anxiety and 20% depression. This study further linked parent mental health to child neurodevelopmental outcomes, demonstrating that higher parental stress correlated with lower child cognitive scores and that severe types of CHD were associated with poorer language development in children.

Our study’s results underscore the need for comprehensive, family-centered care approaches in pediatric cardiology. Interventions focusing on psychoeducation, stress management, and peer support groups could facilitate improved emotional resilience and reduce parental burnout. Combining such strategies with regular medical evaluations might promote not only better outcomes for children but also healthier, more resilient family units. Future research should investigate the long-term sustainability of these improvements, potential differences across cultural backgrounds, and the comparative effectiveness of targeted psychosocial interventions vs. usual care. Moreover, identifying optimal time points for intervention—perhaps shortly after a new diagnosis or following an acute medical event—may maximize the benefits for families grappling with prolonged uncertainty.

### 4.2. Study Limitations

Despite its contributions, this study has several limitations. First, our sample size, though sufficient for preliminary findings, may limit the generalizability of results. Larger, multicenter studies would be beneficial to validate our observations and capture a broader representation of familial circumstances. Second, while we employed well-established instruments (SF-36, PSS, and PBA), these measures rely on self-reporting, which can introduce response and recall bias. Third, the single follow-up point at six months may not fully capture the dynamic, evolving nature of both the child’s clinical condition and the caregiver’s psychological state. Additionally, there is a higher than average proportion of participants with a university degree than the national average in Romania [25], potentially creating a selection bias. Lastly, we did not incorporate an in-depth qualitative component that could enrich our understanding of caregivers’ lived experiences and coping mechanisms. Future investigations combining quantitative and qualitative methods may better illuminate the complexities of caregiver well-being.

## 5. Conclusions

This study provides important insights into the multifaceted experiences of caregivers managing children with congenital cardiac malformations under conservative treatment. Our findings reveal significant, but modest, improvements in physical and mental quality of life, alongside measurable reductions in stress and parental burnout over a six-month period. Notably, subgroup analyses highlight that caregivers of children with mild or moderate malformations and female caregivers may derive more pronounced psychosocial benefits, suggesting that interventions might need to be tailored based on both clinical and demographic factors. Moreover, the exact drivers of these improvements remain multifactorial, ranging from psychological acclimatization to enhanced social support. By addressing both the medical and psychosocial dimensions of conservative cardiac management, healthcare providers can better support families, thereby improving overall outcomes for children. Future studies with larger cohorts and control groups, extended follow-up intervals, and mixed-methods designs are warranted to validate and expand upon these findings, further guiding family-centered interventions and resource allocation in pediatric cardiology settings.

## Figures and Tables

**Table 1:** Demographic and clinical characteristics of the final cohort.

Variable	Value
Caregivers (n)	78
Caregiver Age, years (median, IQR)	36 (30–42)
Caregiver Gender	56 females (71.8%), 22 males (28.2%)
Education	High school: 31 (39.7%)
	Higher education: 47 (60.3%)
Child Age, months (median, IQR)	6.0 (5.5–6.5) *
Child Gender	34 females (43.6%), 44 males (56.4%)
Malformation Severity	Mild: 24 (30.8%)
	Moderate: 38 (48.7%)
	Severe: 16 (20.5%)
Employment Status	Employed: 52 (66.7%)
	Unemployed: 26 (33.3%)
Types of Cardiac Malformations	Patent ductus arteriosus: 14 (17.9%)
	Ventricular septal defect: 20 (25.6%)
	Atrial septal defect: 16 (20.5%)
	Coarctation of the aorta: 12 (15.4%)
	Tetralogy of Fallot: 10 (12.8%)
	Pulmonary stenosis: 6 (7.7%)

*—time of baseline measurement.

**Table 2:** Baseline vs. follow-up SF-36 scores.

SF-36 Domain	Baseline Mean (SD)	6-Month Mean (SD)	*p*-Value
Physical Functioning	62.3 (12.4)	66.7 (12.9)	0.034
Physical Role	54.8 (14.1)	58.2 (14.7)	0.091
Bodily Pain	61.9 (13.0)	65.1 (13.3)	0.084
General Health	58.7 (12.8)	62.8 (13.9)	0.044
Vitality	59.1 (10.6)	63.2 (11.7)	0.028
Social Functioning	64.6 (11.7)	67.3 (12.6)	0.108
Emotional Role	56.2 (13.2)	59.4 (13.8)	0.074
Mental Health	60.8 (12.0)	64.2 (12.6)	0.036
Physical Component Summary	59.7 (11.7)	63.5 (12.1)	0.026
Mental Component Summary	58.3 (12.3)	61.5 (13.1)	0.056

SF—short form; SD—standard deviation; *p*-values not adjusted to test multiple dimensions of SF-36.

**Table 3:** Baseline vs. follow-up Perceived Stress Scale (PSS) scores.

PSS Item	Baseline Mean (SD)	6-Month Mean (SD)	*p*-Value
Total PSS (0–40 scale)	22.9 (6.2)	20.4 (5.9)	0.012

PSS—Perceived Stress Scale; SD—standard deviation.

**Table 4:** Baseline vs. follow-up Parental Burnout Assessment (PBA) scores.

PBA Domain	Baseline Mean (SD)	6-Month Mean (SD)	*p*-Value
Exhaustion in Parenting	15.2 (3.1)	13.9 (2.9)	0.021
Emotional Distancing	14.1 (3.5)	12.8 (3.2)	0.009
Loss of Parental Fulfillment	15.6 (3.4)	13.4 (3.3)	0.004
Total PBA	44.9 (8.5)	40.1 (8.0)	0.003

SD—standard deviation; PBA—Parental Burnout Assessment.

**Table 5:** Correlations among SF-36 (PCS, MCS), PSS, and PBA at follow-up.

Variable	PCS	MCS	PSS	PBA
PCS (SF-36)	1	0.52 *	−0.46 *	−0.40 *
MCS (SF-36)	0.52 *	1	−0.58 *	−0.52 *
PSS	−0.46 *	−0.58 *	1	0.60 *
PBA (Total)	−0.40 *	−0.52 *	0.60 *	1

*—statistically significant (*p*-value < 0.05).

**Table 6:** Subgroup analysis by cardiac malformation severity and caregiver gender.

	n	ΔSF-36 PCS (Mean ± SD)	ΔPSS (Mean ± SD)	ΔPBA (Mean ± SD)	*p*-Value PCS	*p*-Value PSS	*p*-Value PBA
Mild Malformation, Female Caregivers	12	+4.5 (3.6)	−2.8 (2.6)	−5.0 (5.3)	0.04	0.019	0.01
Mild Malformation, Male Caregivers	12	+4.1 (3.8)	−2.6 (2.4)	−4.8 (5.1)	0.05	0.025	0.012
Moderate Malformation, Female Caregivers	19	+3.3 (3.4)	−2.2 (2.3)	−4.3 (4.7)	0.042	0.034	0.026
Moderate Malformation, Male Caregivers	19	+3.1 (3.6)	−2.0 (2.5)	−4.1 (4.5)	0.049	0.04	0.029
Severe Malformation, Female Caregivers	8	+2.5 (3.1)	−1.7 (2.2)	−3.1 (4.2)	0.085	0.074	0.062
Severe Malformation, Male Caregivers	8	+2.3 (3.3)	−1.5 (2.4)	−2.9 (3.9)	0.097	0.083	0.07
Female Caregivers (All Severities)	56	+3.8 (3.5)	−2.5 (2.4)	−4.2 (4.5)	0.031	0.014	0.008
Male Caregivers (All Severities)	22	+2.7 (3.0)	−1.9 (2.2)	−3.1 (4.0)	0.084	0.056	0.049

ANCOVA models with interactions and adjustment for baseline values.

## Data Availability

Data available on request.
